# Monitoring of Putative Vectors of Bluetongue Virus Serotype 8, Germany

**DOI:** 10.3201/eid1509.090562

**Published:** 2009-09

**Authors:** Bernd Hoffmann, Burkhard Bauer, Christian Bauer, Hans-Joachim Bätza, Martin Beer, Peter-Henning Clausen, Martin Geier, Jörn M. Gethmann, Ellen Kiel, Gabriele Liebisch, Arndt Liebisch, Heinz Mehlhorn, Günter A. Schaub, Doreen Werner, Franz J. Conraths

**Affiliations:** Friedrich-Loeffler-Institut, Greifswald-Insel Riems, Germany (B. Hoffmann, M. Beer); Free University of Berlin, Berlin, Germany (B. Bauer, P.-H. Clausen); Justus Liebig University Giessen, Giessen, Germany (C. Bauer); Federal Ministry for Food, Agriculture and Consumer Protection, Bonn, Germany (H.-J. Bätza); University of Regensburg, Regensburg, Germany (M. Geier); Friedrich-Loeffler-Institut, Wusterhausen, Germany (J.M. Gethmann, F.J. Conraths); Carl von Ossietzky University Oldenburg, Oldenburg, Germany (E. Kiel); Zecklab, Burgwedel, Germany (G. Liebisch, A. Liebisch); Heinrich-Heine-University of Düsseldorf, Düsseldorf, Germany (H. Mehlhorn); Ruhr University, Bochum, Germany (G.A. Schaub); Leibniz-Center for Agricultural Landscape Research e. V, Müncheberg, Germany (D. Werner)

**Keywords:** Culicoides, bluetongue disease, bluetongue virus, monitoring, epidemiology, RT-PCR, viruses, Germany, dispatch

## Abstract

To identify the vectors of bluetongue virus (BTV) in Germany, we monitored *Culicoides* spp. biting midges during April 2007–May 2008. Molecular characterization of batches of midges that tested positive for BTV suggests *C. obsoletus* sensu stricto as a relevant vector of bluetongue disease in central Europe.

Bluetongue disease (BT), discovered north of the Alps in Europe in August 2006 (*1*–*5*), causes massive losses of farm ruminants, particularly sheep. Epidemic BT has been caused by serotype 8 of bluetongue virus (BTV-8). The virus overwintered and spread over a large area in 2007 (*5*,*6*). *Culicoides* spp. biting midges can transmit BT. In the Mediterranean region, BT is mainly transmitted by *C. imicola* midges, a species that has so far not been detected north of the Alps (*7*,*8*). We aimed to determine the abundance of hematophagous *Culicoides* spp. biting midges and to identify putative vectors of BTV in Germany.

## The Study

**Figure 1:**
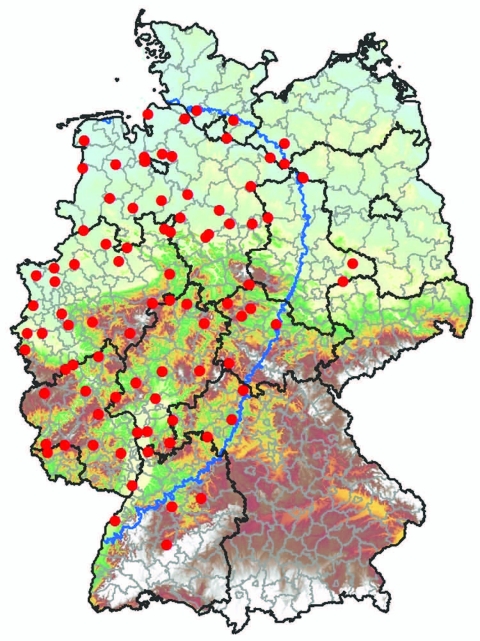
*Culicoides* spp. monitoring area, 150-km zone restricted because of the occurrence of bluetongue disease in Germany as of January 2007 (blue border), and geographic positions of 89 black light traps (red dots).

Biting midges were caught from April 2007 through May 2008 by using 89 black light traps (BG-Sentinel Trap; Biogents, Regensburg, Germany) distributed mostly in the German BT restriction zone of January 2007 (Figure 1). Most (n = 85) traps were placed in the vicinity of cow sheds, either adjacent to barns or in their entrance area; 4 additional traps were placed in pastures if the cattle were kept there day and night.

Biting midges were caught during 7 consecutive nights in the first week of each month. We preserved them in 70% ethanol and sorted them under the dissection microscope according to wing patterns (*9*,*10*) into batches of <50 parous, nulliparous, or blood-fed female or male insects of the *C. obsoletus* group (including *C. dewulfi*), the *C. pulicaris* group, or other *Culicoides* spp. biting midges.

Batches of female biting midges were tested for BTV by real-time reverse transcription–PCR (rRT-PCR). Each batch was homogenized in 400 μL lysis buffer (NucleoSpin 96 Virus kit; Macherey-Nagel, Düren, Germany) with a 5-mm steel bead by using a TissueLyser instrument (QIAGEN, Hilden, Germany) for 2 min at 30 Hz. After short centrifugation at 12,000 × *g*, nucleic acids were extracted from 200 µL homogenate (NucleoSpin 96 Virus kit) on a Tecan Freedom EVO automatic platform (Tecan Deutschland GmbH, Crailsheim, Germany). RNA was analyzed by using the iScript One-Step RT-PCR Kit for Probes (Bio-Rad, Munich, Germany) in a duplex rRT-PCR (pan-BTV-duplex rRT-PCR) combining a BTV rRT-PCR that detects all known serotypes (*11*) with a PCR that detects all members of the genus *Culicoides* (pan-*Culicoides* assay) as an internal control for RNA extraction and amplification. For the pan-*Culicoides* assay, primers and a probe were selected from aligned sequences of the rDNA internal transcribed spacer 1 and 2 (Pan-Culi-ITS1+2-597F [5′-CAG GAC ACA CGA TCA TTG ACA-3′], Pan-Culi-ITS1+2-976R [5′-CAC ATG AGY TGA GGT CGT CAT-3′], Pan-Culi-ITS1+2-623HEX [5′-HEX-AAC GCA TAT TGC ACC CCA TGC GA-BHQ1-3′]). To confirm positive pan-BTV-duplex rRT-PCR results, we extracted RNA from the remaining homogenate of the batch and subjected it to BTV-8- rRT-PCR (*5*). Batches were considered BTV positive if results of both assays were positive. Batches of the *C. obsoletus* and *C. pulicaris* groups with high viral loads were further analyzed for *Culicoides* spp. by amplification of the mitochondrial cytochrome oxidase subunit I (*12*).

**Figure 2:**
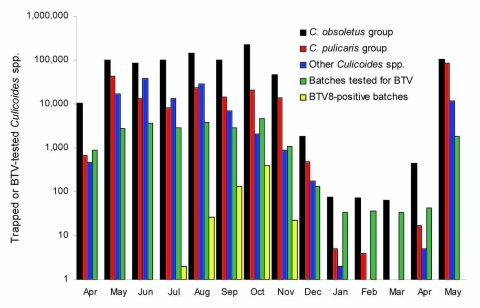
Monthly catches of midges of the *Culicoides obsoletus* group (black), *C. pulicaris* group (red), and other *Culicoides* spp. (blue) captured with 89 black light traps in Germany during 7 consecutive nights in the first week of each month during the study period (April 2007–May 2008). Batches consisting of <50 female biting midges were tested for bluetongue virus (BTV) by real-time reverse transcription–PCR. The total number of batches (green) and the number of batches positive for BTV (gold) are shown.

The overall number of biting midges caught started at a moderate level (11,577) in April 2007, peaked in October (246,882), decreased to low levels during December 2007–March 2008, and started to rise again (462) in April 2008 (Figure 2). Small numbers (66–81) of *Culicoides* spp. midges also were detected in some traps during January–March 2008. Members of the *C. obsoletus* group (including *C. dewulfi*) were most frequently trapped, followed by the *C. pulicaris* group. Biting midges of the *C. pulicaris* group were more often collected during spring and summer 2007 in discrete locations.

Of 24,513 batches analyzed by rRT-PCR, 16,206 (66.1%) batches belonged to the *C. obsoletus* group, 5,796 (23.6%), to the *C. pulicaris* group, and 2,511 (10.2%) to other *Culicoides* spp. A total of 585 (2.4%) batches were positive for BTV by rRT-PCR, 562 (96.1%) of which belonged to the *C. obsoletus* group, 16 (2.7%), to the *C. pulicaris* group, and 6 (1.0%) to other *Culicoides* spp.; 1 was identified as *C. achrayi*. The number of positive pools varied considerably by month (Figure 2). All batches that were positive in the pan-BTV-rRT-PCR analysis with a cycle threshold (Ct) value <37 (n = 464) were confirmed as BTV positive. BTV-infected biting midges (*C. pulicaris* group) were first detected in June 2007, a few weeks after the first new infection of the year with BTV-8 had been discovered (*5*). The number of BTV-positive *Culicoides* batches peaked (n = 401) in October 2007, which coincided with the peak of midge abundance (Figure 2). During December 2007–May 2008, no BTV-positive batches were detected.

A total of 540 batches of biting midges carried a low or medium (Ct values >30–40), 38 a high (Ct values 25–30), and 7 a very high (Ct values <25) BTV genome load. Batches with a high virus genome load showed Ct values similar to that of highly positive, undiluted blood samples from cattle or sheep. Although ≈70–100 µL of cattle or sheep blood are used for the BTV genome detection, <1 µL blood remains in a biting midge after a blood meal. The uptake of highly BTV-positive blood can therefore only lead to a Ct value increased at least by 6 or 7 when the biting midge is tested by rRT PCR for BTV. Our findings provide strong evidence for virus replication in the biting midges in the highly positive pools.

All batches with very high and 36 batches with a high virus genome load consisted of midges of the *C. obsoletus* group. Only 2 batches with a high viral load belonged to the *C. pulicaris* group. These data clearly support the previously suggested role of species of the *C. obsoletus* group as competent vectors for BTV (*13*,*14*).

The species composition of batches of the *C. obsoletus* and *C. pulicaris* groups with a high viral genome load was further determined by PCRs of the mitochondrial cytochrome oxidase subunit I (*10*). Although our analyses showed that most batches consisted of several species (Table 1), *C. obsoletus* sensu stricto was identified in all investigated batches of the *C. obsoletus* group (Table 1). Furthermore, 18 BTV-positive batches morphologically classified as *C. obsoletus* group consisted exclusively of *C. obsoletus* sensu stricto. These findings indicate that *C. obsoletus* sensu stricto is involved in the transmission of BTV in Germany, but a role for other members of the *C. obsoletus* group in the transmission of BTV cannot be ruled out. In contrast, the characterization of the pools of the *C. pulicaris* group yielded inconclusive results (Table 2). More than 1 species-specific fragment was amplified in all tested pools, but *C. punctatus* could be identified in all investigated pools.

**Table 1:** Genetic characterization of batches of midges of the *Culicoides obsoletus* group, Germany, April 2007–May 2008*

Batch no.	rRT-PCR, Ct	*C. obsoletus sensu stricto*	*C. scoticus*	*C. chiopterus*	*C. dewulfi*
270/38F	20.10	+++	–	+++	+++
276/2T	20.74	+++	–	+++	+++
306/12FN	21.33	+++	–	+++	+++
304/34B	21.60	+++	–	+++	–
276/2O	23.40	+++	–	+++	+++
296/52Q	23.87	+++	–	+++	+++
296/44G	24.00	+++	–	+++	+++
296/22R	25.90	+++	–	+++	+++
296/52AL	27.26	+++	–	+++	+++
306/1X	27.30	+++	–	–	–
306/1A	27.60	+++	–	–	–
306/1E	27.70	+++	–	–	–
306/1Z	27.70	+++	–	–	–
270/45B	27.76	+++	–	–	+++
306/1L	27.90	+++	–	–	–
306/1T	27.90	+++	–	–	–
263/46B	28.00	+++	–	++	+++
306/1H	28.00	+++	–	–	–
306/1AA	28.00	+++	–	–	–
306/1S	28.10	+++	–	–	–
306/1AC	28.10	+++	–	–	–
18/156B	28.18	+++	–	+	+++
346/29C	28.19	+++	–	–	+
306/1R	28.30	+++	–	–	–
306/1V	28.30	+++	–	–	+++
306/1N	28.40	+++	++	–	–
276/10S	28.41	+++	–	+++	+++
306/1I	28.50	+++	–	–	–
306/1M	28.70	+++	+	+++	–
263/46A	28.80	+++	–	+++	–
296/51AH	28.82	+++	++	+++	+++
306/1D	28.90	+++	–	–	–
171/13E	29.00	+++	–	–	+++
306/1C	29.00	+++	+++	–	–
304/1H	29.10	+++		+++	+++
306/1B	29.20	+++	–	–	–
306/1F	29.20	+++	–	–	–
306/1U	29.20	+++	–	–	–
336/24B	29.21	+++	–	+++	–
306/12LR	29.26	+++	–	+++	+++
306/1K	29.30	+++	–	–	–
306/1J	29.40	+++	–	–	–
270/55I	29.47	+++	–	+++	+++

*rRT-PCR, real-time reverse transcription–PCR; Ct, cycle threshold; –, no visible band; +, faint; ++, distinct; +++, strong band after agarose gel electrophoresis of PCR products and staining with ethidium bromide.

**Table 2:** Genetic characterization of batches of midges of the *Culicoides pulicaris* group, Germany, April 2007–May 2008*

Batch no.	rRT-PCR, Ct	*C. pulicaris* s.s.	*C. punctatus*	*C. impunctatus*	*C. grisescens*	*C. newsteadi*
263/49 A	28.57	–	++	+	+	–
263/49 B	29.46	+++	+++	++	+	+
276/46B	31.37	–	+++	–	+	+++
276/62A	34.43	+++	+++	–	–	–
292/4A	33.97	+++	+++	–	–	–
304/63A	32.90	+++	+++	+	–	++

*rRT-PCR, real-time reverse transcription–PCR; Ct, cycle threshold; –, no visible band; +, faint; ++, distinct; +++, strong band after agarose gel electrophoresis of PCR products and staining with ethidium bromide.

## Conclusions

Our study yielded no evidence that *C. imicola* midges occurred in the study area in Germany. Members of the *C. obsoletus* group were detected in the entire monitoring area in high abundances and frequently contained BTV genome. Because of the detection of BTV in a high number of batches, which consisted of *C. obsoletus* sensu stricto, this species must be assumed to play a major role as a vector of BTV in Germany.
